# Vascular effects of deletion of melanocortin-4 receptors in rats

**DOI:** 10.1002/phy2.146

**Published:** 2013-11-13

**Authors:** David W Stepp, Christabell C Osakwe, Eric J Belin de Chantemele, James D Mintz

**Affiliations:** 1 Vascular Biology Center, Georgia Regents UniversityAugusta, Georgia; 2 Department of Physiology, Georgia Regents UniversityAugusta, Georgia

**Keywords:** Blood pressure, obesity, vascular

## Abstract

Obesity is a major cause of hypertension, but links between the obese and hypertensive states remain incompletely understood. A major component of cardiovascular function in obese individuals is a state of sympathoactivation. A postulated mechanism of this sympathoactivation is the activation of specific classes of neurons commonly associated with metabolic control, which also affect sympathetic outflow to cardiovascular targets. One class of neurons is characterized by expression of melanocortin-4 receptors (MC4R) which are activated by metabolic signals such as leptin and insulin. In this study, we examined the effects of deletion of MC4R in a novel rat model. MC4R knockout (KO) rats are obese and profoundly insulin resistant without frank diabetes. Despite these conditions, MC4R KO rats are normotensive. Moderate bradycardia and significant increases in peripheral resistance were evident in MC4R KO rats. To determine if the dissociation between hypertension and obesity was associated with changes in vascular function, in vitro reactivity to vasoactive agents and in vivo reactivity to sympathetic blockade were examined. Vasodilator function was not affected by obesity in MC4R KO rats. Reactivity to phenylephrine was reduced, suggesting desensitization of adrenergic signaling. In response to ganglionic blockade with mecamylamine, blood pressure and hindlimb resistance fell more in MC4R KO rats, suggesting that sympathoactivation of the vascular was still evident, despite the absence of hypertension. These findings suggest that obesity causes sympathoactivation of the vasculature despite the absence of MC4R. Dissociation of obesity from hypertension in this model may reflect more renal mechanisms of blood pressure control.

## Introduction

Obesity is one of the major causes of hypertension in Western cultures, but links between the obese and hypertensive states remain incompletely understood. The prevailing consensus is that a sympathoactivated state common in obesity is a major contributor to impaired blood pressure regulation (Hall et al. 1999), but precise links are not universally accepted. Links to metabolism such as leptin (Hall et al. 2001) or insulin (Scherrer and Sartori 1997) have been demonstrated to provoke sympathetically mediated cardiovascular responses such as increases in arterial pressure and sympathetic nerve activity, but these results are confounded by evidence of elevated blood pressure in leptin-deficient models (Alonso-Galicia et al. 1996) and indications that sympathoactivation can be thwarted by adrenergic desensitization (Belin de Chantemele et al. 2009).

Neuronal targets of leptin and insulin in the hypothalamus further project to neurons expressing melanocortin-4 (MC4R) receptors (da Silva et al. 2004; Tallam et al. 2006; Hall et al. 2010). Activation of these neurons results in stimulation of increases in arterial pressure that are inhibited by adrenergic blockade (Kuo et al. 2004). Genetic defects that interrupt MC4R signaling in humans result in lower blood pressure despite prevailing obesity (Greenfield 2011). Measures of muscle sympathetic nerve activity are reduced in humans (Sayk et al. 2010), but measures of sympathetic activation of the cardiovascular system are incomplete in models of MC4R deletion.

The recent generation of MC4R knockout rats provides a novel tool to explore the relationship between the MC4R receptor, cardiovascular sympathetic tone, and vascular function. While MC4R knockout mice have been available for some time (Huszar et al. 1997), the rat provides a new opportunity to obtain higher resolution phenotypes (longer measurements of blood pressure, more robust hypertension, more easily obtained blood flow measurements) that are difficult to obtain in mice. Accordingly, the goal of this study was to test the hypothesis that MC4R KO impacted cardiovascular control in obesity. This study examined the effects of deletion of MC4R on vascular function. We examined metabolic parameters, long-term blood pressure via telemetry, sympathetic contribution to vascular tone, and reactivity to major physiologic determinants of perfusion.

## Methods

### Animals

Adult male Wild-Type (WT) and MC4R Knockout rats (MC4RKO) generated via targeted chemical mutagenesis at the Hubrecht Institute in the lab of Dr. Edwin Cuppen (Van Boxtel et al. 2011) and licensed and marketed by Transposagen/Taconic were acquired at 10 weeks of age and studied up to 20 weeks of age. All animals were maintained in an AAALAC-approved animal facility and the GHSU Institutional Animal Care and Use Committee approved all procedures.

### Telemetry

Rats were implanted with telemetry transmitters (Data Sciences International, St. Paul, MN) in the abdominal aorta during the week prior to the initiation of the study as described previously (D'Angelo et al. 2006). Rats were isoflurane anesthetized and a midline incision was made to expose the abdominal aorta. The aorta was briefly occluded to allow insertion of the transmitter catheter that was secured in place with tissue glue. The transmitter body was sutured to the abdominal wall along the incision line as the incision was closed. The skin was closed with staples and allowed to recover from surgery before being returned to their home cages. Individual cages were placed on top of the telemetry receivers and arterial pressure wave forms were continuously recorded throughout the study. Data were sampled at 10 min intervals and captured as mean, diastolic and systolic pressure, and heart rate. Pressure variability was assessed as the frequency distribution of each 10 min point for 7 days in six rats.

### Vascular reactivity

Thoracic aorta from MC4R KO and WT rats were surgically dissected and mounted on a wire myograph (DMT, Aarhus, Denmark) with 1 g basal tension. Briefly, two tungsten wires were inserted into the lumen of the arteries and fixed to a force transducer and a micrometer. Arteries were bathed in a physiological salt solution as previously described (Stepp and Tulenko 1994). Arterial viability was determined using a potassium rich solution (40 mmol/L). Vascular contractility was assessed with cumulative concentration–response curves (CRC) to phenylephrine (PE, 1 pmol/L to 10 *μ*mol/L) and serotonin (5HT, 1 pmol/L to 10 *μ*mol/L). Constriction to NE, PE, and 5HT were expressed as percent of KCl-induced constriction. The endothelial function was assessed with CRC to acetylcholine (ACh), and endothelium-independent relaxation was analyzed with a CRC to sodium nitroprusside (SNP, 1 pmol/L to 10 *μ*mol/L). Relaxation curves were performed on preconstricted vessels (PE, 1 *μ*mol/L) and the relaxation expressed as percent of the precontraction.

### Sympathetic contribution to vascular tone

Animals were catheterized for the measurement of mean arterial pressure (MAP, carotid artery) and venous drug delivery (jugular vein) and anesthesia was maintained with isoflurane. A midline incision was performed and the superior mesenteric artery and the distal aorta at the iliac bifurcation were exposed. Adventitial tissue was gently removed and a 1PRB (mesenteric) or 2PS (aorta) Transonic flow probe was placed around the vessel. Acoustic coupling was achieved by a coating of heart rate (HR) conductance jelly. Ganglionic blockade was achieved with 2 mg/kg mecamylamine. Effectiveness of blockade was determined by administration of PE (24 *μ*g/kg) and verifying no reflex drop in heart rate. Blood flows were normalized to organ weight and vascular resistance was calculated as the quotient of pressure over flow (mmHg mL^−1^ min^−1^ g^−1^).

### Statistics

Metabolic parameters, hemodynamic variables, and peak vascular responses were compared between age-matched Control and MC4R KO rats using a Student's *t*-test. A *P* < 0.05 was considered statistically significant. Values are presented as mean ± SEM.

## Results

### Baseline phenotype

Baseline anatomic and metabolic features of the animals used in this study are summarized in Table 1. MC4R KO rats demonstrated hyperphagia and a predictable increase in body weight versus WT controls. Increased body weight was associated with increases in heart and kidney mass, likely reflective of increases in cardiac output and urine flow, respectively. Brain weight and snout-to-anus length were similar between groups.

**Table 1:** Effects of deletion of melanocortin-4 receptors on anatomic, behavioral, and metabolic parameters in rats

	Wild type	MC4R knockout rats
Anatomic		
Body weight (g)	511 ± 45	793 ± 95*
Heart weight	1.38 ± 0.2	1.68 ± 0.2*
Kidney weight	1.72 ± 0.2	2.32 ± 0.3*
Brain weight	2.06 ± 0.2	2.14 ± 0.1
Snout-to-anus length	10.7 ± 1	10.8 ± 1
Behavioral		
Food intake (g/day)	21 ± 1	39 ± 3*
Water intake (mL/day)	28 ± 3	46 ± 3*
Urine output (mL/day)	16 ± 3	25 ± 2*
Activity (counts/day)	2.0 ± 0.2	1.6 ± 0.3
Metabolic		
Cholesterol (mg/dL)	140 ± 13	270 ± 23*
Triglycerides (mg/dL)	107 ± 13	444 ± 49*
NEFA (mg/dL)	0.33 ± 0.01	0.38 ± 0.01*
Glucose (mg/dL)	115 ± 5	121 ± 7
HBA1c%	4.9 ± 0.1	6.0 ± 0.2*
Insulin (ng/mL)	0.24 ± 0.6	1.5 ± 0.5*
Leptin (pg/mL)	1434 ± 101	7029 ± 553*

Data are mean ± SEM of *n* ≥ 8 for each group.

* *P* < 0.05.

The metabolic phenotype of the MC4R KO rat was consistent with obesity-related insulin resistance, including elevations in plasma levels of cholesterol, triglycerides, free fatty acids, HbA1c, and insulin. Frank diabetes was not evidenced as fasting blood glucose was normal between groups. Leptin levels were increased, likely reflecting the combined effects of obesity and loss of the MC4R receptor. Despite adiposity and metabolic dysfunction, activity levels assessed by telemetry were similar between groups. Taken together, these data suggest that the MC4R rat is a model of obesity and insulin resistance in the prediabetic state.

### Conscious hemodynamics

**Figure 1:**
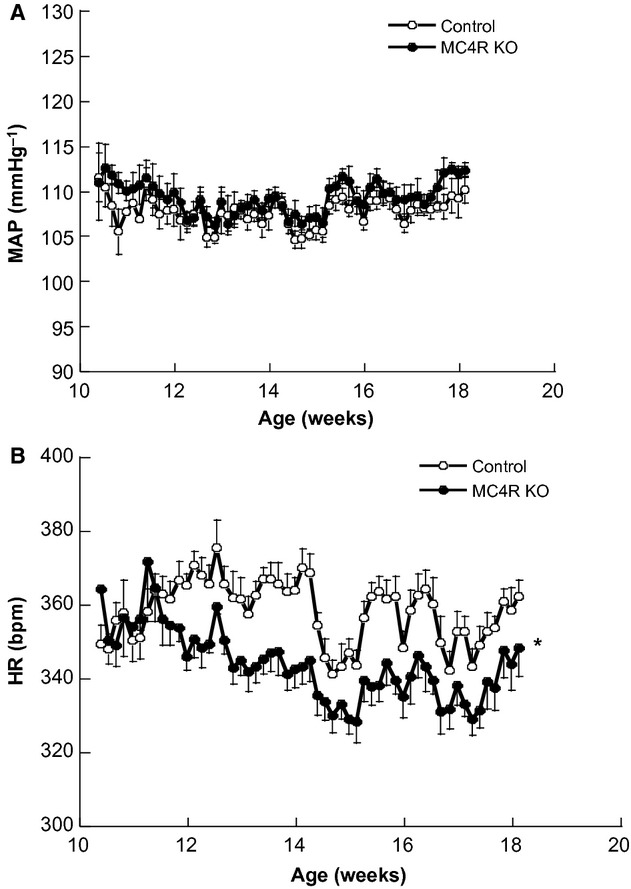
In vivo conscious hemodynamics in MC4R KO and control rats under basal conditions over a period of 8 weeks. (A) is 24 h mean arterial pressure and (B) is 24 h mean heart rate. Data are presented as mean ± SEM (*n* = 6, *P < 0.05).

The effects of MC4R deletion on blood pressure and heart rate in rats is shown in Figure 1. Despite obesity and significant metabolic dysfunction, blood pressure (Fig. 1A) was similar in WT and MC4R KO rats from young adulthood to full maturity (10 through 18 weeks). Consistent with work in MC4R KO mice (Tallam et al. 2005), a significant bradycardia was observed in full adult MC4R KO rats, although younger rats had a normal heart rate. Hemodynamic diurnal rhythms were unaffected by MC4R deletion (Table *).

### Vascular reactivity

**Figure 2:**
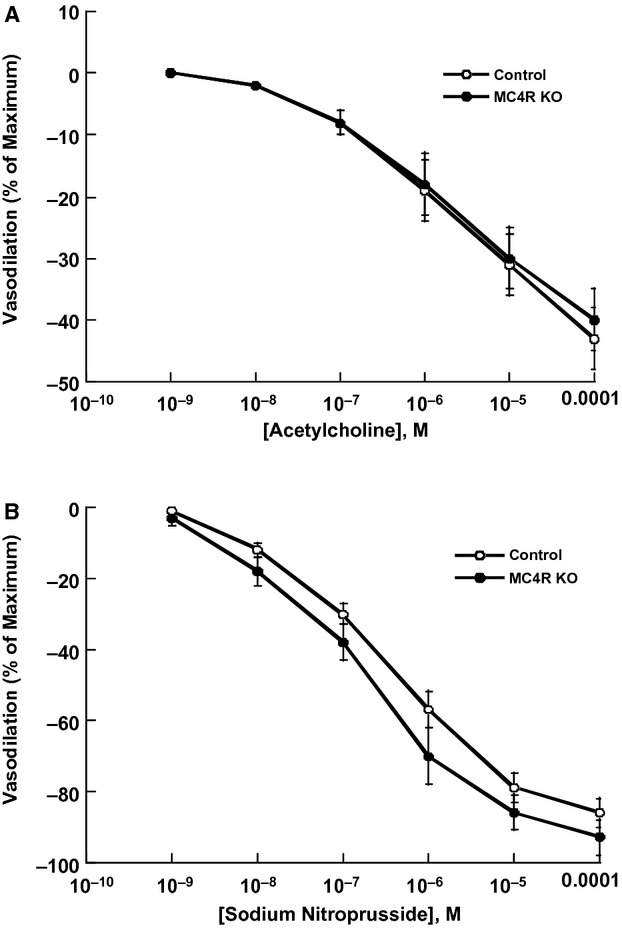
In vitro aortic vasodilator reactivity to the endothelium-dependent vasodilator acetylcholine (A) and to the nitric oxide donor sodium nitroprusside (B) in control and MC4R KO rats. Data are normalized to baseline preconstriction and presented as mean ± SEM (*n* = 6. *P* = NS).

**Figure 3:**
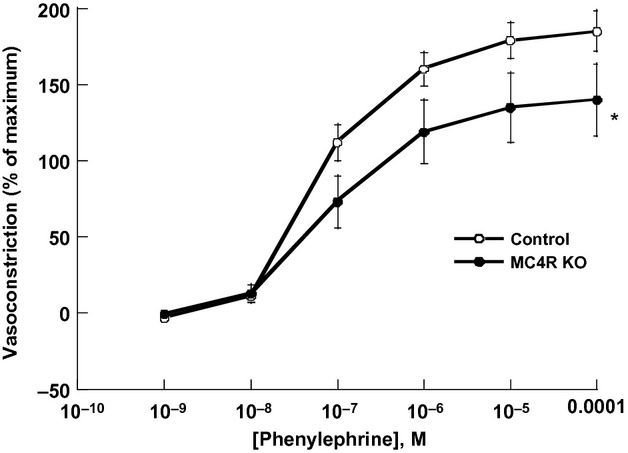
In vitro aortic vasoconstrictor reactivity to the *α*-adrenergic receptor agonist phenylephrine normalized to KCl reference constriction in control and MC4R KO rats. Data are presented as mean ± SEM (*n* = 6. *P < 0.05).

Responsiveness to vasodilator agonists are shown in Figure 2 and vasoconstrictor agonists are shown in Figure 3. Despite significant metabolic dysfunction and obesity, vasodilator activity was similar between groups for ACh (endothelium-dependent, Fig. 2A) and nitroprusside (endothelium-independent, Fig. 2B) vasodilation. Vasoconstrictor responses to KCl were moderately elevated (1.2 ± 0.1 vs. 1.7 ± 0.2 g of force, Wild Type vs. MC4R KO, *P* < 0.05, *n* = 6) and normalized to KCL, serotonin reactivity was similar (198 ± 5 vs. 189 ± 22%, Wild Type vs. MC4R KO, *P* = NS *n* = 6). Adrenergic reactivity to PE was significantly reduced in MC4R KO rats (Fig. 3 and Table 2).

**Table 2:** Telemetric hemodynamic data during week 18 of life of WT and MC4RKO rats

	Wild type	MC4R knockout rats
Arterial pressure (mmHg)		
Mean (24 h)	108 ± 1	111 ± 1
Diastolic (24 h)	92 ± 1	94 ± 1
Systolic (24 h)	129 ± 1	130 ± 1
Mean (diurnal)	110 ± 3	107 ± 1
Mean (nocturnal)	111 ± 1	114 ± 1
Heart rate	354 + 4	340 + 6*

Data are mean **±** SEM of *n* ≥ 5 for each group.

* *P* < 0.05.

### Evidence of sympathetic activation of the vasculature

**Figure 4:**
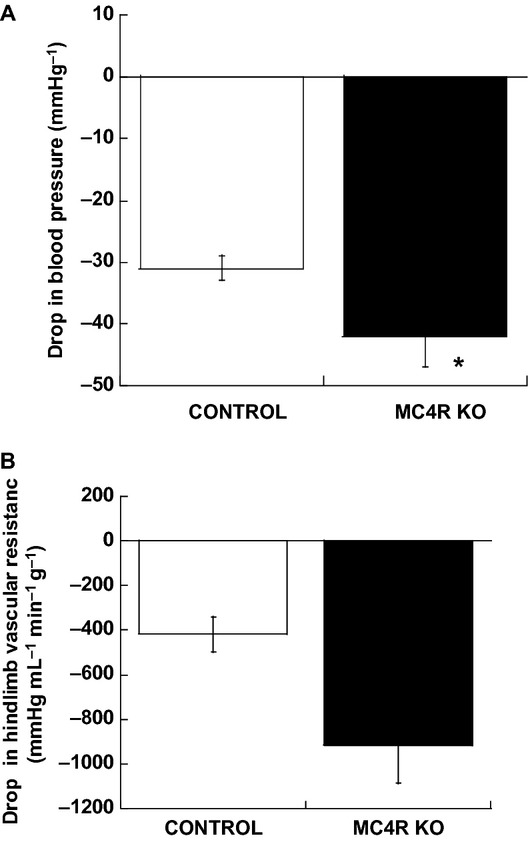
In vivo hemodynamic responses to ganglionic blockade with mecamylamine (2 m/kg). (A) is drop in blood pressure in mmHg. (B) is the drop in hindlimb vascular resistance and mmHg mL^−1^ min^−1^ g^−1^. Data are presented as mean ± SEM (*n* ≥ 8. *P < 0.05).

To assess sympathetic control of vasomotor tone, anesthetized rats were used to measure hemodynamic reactivity to ganglionic blockade. Under isoflurane anesthesia, blood pressure was similar between groups (101 ± 6 vs. 106 ± 5, *n* ≥ 8, *P*=NS) and vascular resistance was significantly increased in the mesenteric (98 ± 9 vs. 140 ±16 mL min^−1^ g^−1^ mmHg^−1^, *P* < 0.05, *n* ≥ 8) and hindquarters (1005 ± 103 vs. 1689 ± 199 mL min^−1^ g^−1^mmHg^−1^, *P* < 0.05, *n* ≥ 8) circulations. When sympathetic outflow was interrupted with mecamylamine, the drop in blood pressure was significantly greater (Fig. 4A) in rats lacking MC4R. In parallel, hindquarters resistance dropped 118% more in MC4R KO rats compared with controls (Fig. 4B), such that after ganglionic blockade, resistance was not significantly different (586 ± 60 vs. 739 ± 87 mL min^−1^ g^−1^ mmHg^−1^, *P* = NS, *n* ≥ 8). Heart rate fell 29 ± 11 bpm in MC4R KOs, but had no effect in WT rats (*P* < 0.05).

## Discussion

This study explored the effects of whole-body deletion of MC4R receptors on cardiovascular function. The key findings of this study are (1) despite morbid obesity, rats lacking MC4R receptors are not hypertensive, (2) despite obesity and severe insulin resistance, rats lacking MC4R show no deficits in endothelial dysfunction, and (3) deletion of MC4R produced a significant bradycardia and increased sympathetic tone to the vasculature. Relevant to these findings are comparison to other models, the relationship between endothelial function and obesity and the relationship between sympathetic outflow, obesity, and bradycardia.

### Insights from other models of MC4R receptors deletion

In this study, we find that blood pressure is unchanged and heart rate is lower in MC4R knockout rats. In 2005, Tallam and coworkers 2005 observed similar findings in mice harboring a genetic deletion of the MC4R, indicating that our findings were not limited to rats. Moreover, the reduction in heart rate and dissociation between obesity and hypertension has been found in humans (Greenfield et al. 2009), suggesting that the rat model retains clinically relevant features of MC4R deficiency.

In this study, we extend these findings to novel observations regarding sympathetic outflow to the vasculature. Obesity has been indicated to result in an increase in sympathetic outflow to the circulation. Interruption of sympathetic outflow in Obese Zucker rats results in larger decreases in arterial pressure (Schreihofer et al. 2004), an acute effect that primarily represents a loss of peripheral vascular tone. Moreover, chronic sympathetic stimulation of the vasculature results in a loss of adrenergic reactivity in innervated beds, notably the mesenteric circulation (Romanko and Stepp 2005) and the aorta (Belin de Chantemele et al. 2009). In MC4R KO rats, the larger drop in blood pressure is preserved and aortic reactivity to PE is reduced. These data indicate that that link between obesity and vascular sympathoactivation bypasses the MC4R receptor pathway. In contrast, previous work examining sympathetic activation in obese d*b/*d*b* mice indicated an increase in renal sympathetic nerve activity (RNSA) with leptin-deficient obesity, but a dissociation of obesity and RNSA in MC4R KO mice (Rahmouni et al. 2003). Thus, in terms of sympathetic activation, the relationship between obesity and an increase in MAP best tracks with innervation of the kidney rather than the vasculature. The impact of vascular sympathoactivation in the hypertensive process is less clear.

### The relationship between endothelial function and obesity

As the discovery that obesity impairs endothelial function, the link between the two has been the subject of intense debate. In this study, we observe that obesity induced by deletion of MC4R receptors produced severe obesity and profound insulin resistance, easily in the range of obese models associated with impaired endothelium-dependent vasodilation. While the data from our studies do not include all vascular beds or resistance vessels, the failure to see depressed endothelial function in this study begs the question: Is the cardinal defect in obesity simply hypertension? Recent studies correcting insulin resistance in mice with deletion of protein tyrosine phosphatase 1B implied that insulin resistance drives endothelial dysfunction (Ali et al. 2009), but later studies indicated that the same maneuver also corrected deficits in the control of blood pressure (Belin de Chantemele et al. 2012). The data in the current studies parallel recent studies from do Carmo and colleagues indicating that, like endothelial function, renal injury was limited in MC4R mice even after a lifetime of obesity (do Carmo et al. 2009) and work from our own lab that indicates that stroke damage was entirely corrected by a diuretic antihypertensive in obese Zucker rats, despite no improvement in metabolic status (Osmond et al. 2010). Indeed, the only model in our experience which imposes an endothelial dysfunction and metabolic defect in the absence of hypertension is fructose feeding (D'Angelo et al. 2005; Romanko et al. 2009) suggesting that findings in that model may reflect the artificial processes involved in its generation rather than metabolic dysfunction as manifest by normal disease processes. While it is likely simplistic to assume the hypertension is the sole drive of vascular dysfunction in obesity, this study adds to a growing body of evidence that it may be the primary one.

### The relationship between sympathetic outflow, obesity, and bradycardia

One of the earliest known cardiovascular effects of MC4R stimulation is an increase in heart rate (Van Bergen et al. 1995) and thus the decrease in heart rate in MC4R KO models has been assumed to reflect the loss of MC4R on cardiac rhythm. In this study, we find that sympathetic outflow to the vasculature is increased in MC4R KO rats, as indicated by larger drops in blood pressure and hindlimb vascular resistance. These data complicate the simple interpretation of reduced heart as an increase in peripheral vascular resistance would reflexively result in reduced heart rate via baroreceptor mechanisms to maintain constant blood pressure. Moreover, in young adulthood, heart rate is normal suggesting that the simple MC4R deletion does not result in a reduction in heart rate. Perhaps, the simplest interpretation of this data is that, independent of the MC4R receptor, obesity produces a progressive increase in vascular sympathetic tone that is offset by baroreflex-mediated adjustments in heart rate. Should baroreflexes become impaired (as seen in other models of obesity (Schreihofer et al. 2007) impaired control of arterial pressure would ensue.

Taken together, the data derived from these studies extends our understanding of the role of MC4R in two ways. First, it provides confirmation of the essential cardiovascular feature of MC4R deletion is a new model – dissociation of obesity and hypertension in the rat. Second, it provides new insights into how sympathetic control of cardiovascular system is modulated in obesity and the role, or lack thereof, of MC4R in vascular sympathoactivation. How obesity results in the well-documented increase in vascular sympathetic tone remains to be determined.
